# The only species of *Mohnia* Friele, 1879 (Caenogastropoda, Buccinoidea, Buccinidae) in the North Pacific represents an unrecognized new genus of Newtoniellidae (Triphoroidea)

**DOI:** 10.3897/zookeys.1055.68911

**Published:** 2021-08-05

**Authors:** Ellen E. Strong, Boris I. Sirenko, James H. McLean

**Affiliations:** 1 National Museum of Natural History, Smithsonian Institution, PO Box 37012, MRC 163, Washington, DC 20013-7012, USA Smithsonian Institution Washington United States of America; 2 Zoological Institute, Russian Academy of Sciences, St. Petersburg, 199034, Russia Zoological Institute, Russian Academy of Sciences St. Petersburg Russia; 3 Deceased; formerly of Natural History Museum of Los Angeles County, 900 Exposition Blvd, Los Angeles, CA 90007, USA Natural History Museum of Los Angeles County Los Angeles United States of America

**Keywords:** Anatomy, radula morphology, shell morphology, systematics

## Abstract

*Mohniakurilana* Dall, 1913 was described more than 100 years ago from deep waters off the Kuril Islands and remains exceedingly rare in museum collections. Originally placed in the carnivorous neogastropod family Buccinidae, fragmentary soft parts from the type lot and from several specimens belonging to allied species collected in the Aleutian Islands in the 1990s have allowed anatomical investigations for the first time. These have revealed the presence of a paucispiral operculum with an eccentric nucleus, foot with a deep propodial pedal gland and metapodial pedal gland, taenioglossate radula, short acrembolic proboscis, well-developed mid-esophageal gland, glandular prostate, and the absence of a penis; the nervous system is epiathroid with a long supra-esophageal connective and numerous statoconia in the statocysts. Analysis of the gut contents revealed abundant halichondriid sponge spicules. This evidence indicates a placement in the Triphoroidea, a diverse superfamily of specialized spongivores. *Mohniakurilana* is transferred to the Newtoniellidae and placed in the new genus *Pseudomohnia***gen. nov.***Pseudomohniarogerclarki***sp. nov.** is established for a new species from the Aleutian Islands characterized by its narrowly turreted shell and distinctive multicuspid rachidian. A lectotype is designated for *Mohniakurilana*.

## Introduction

**Figure 1.**
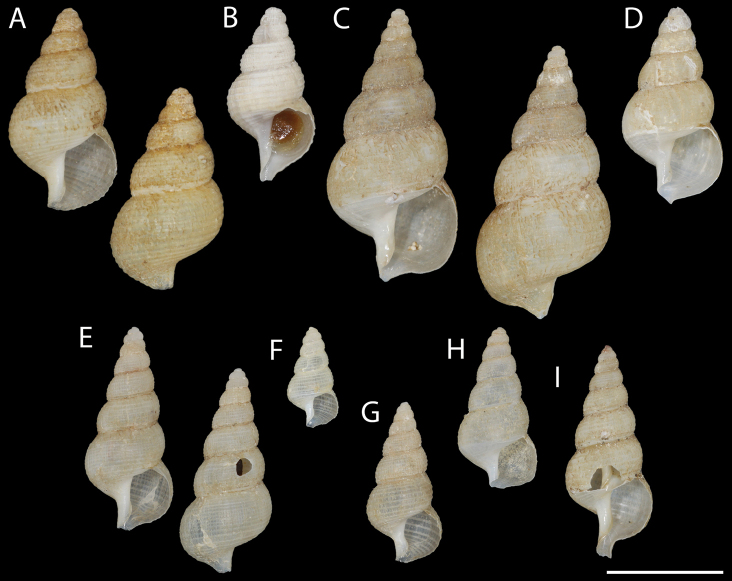
Shell morphology of *Pseudomohnia* gen. nov. **A, B***Pseudomohniakurilana* (Dall, 1913) comb. nov **A** lectotype, USNM 205224. Kuril Islands **B**ZIN 57494/1. Kuril Islands **C, D***Pseudomohnia* sp. LACM 1997-156.7 Aleutian Islands **E–I***Pseudomohniarogerclarki* sp. nov. Aleutian Islands **E** holotype, LACM 3776 **F**LACM 1997-174 **G**LACM 1997-174 **H**LACM 1997-163.21 **I**LACM 1997-165.20. Scale bar: 1 cm.

**Figure 2.**
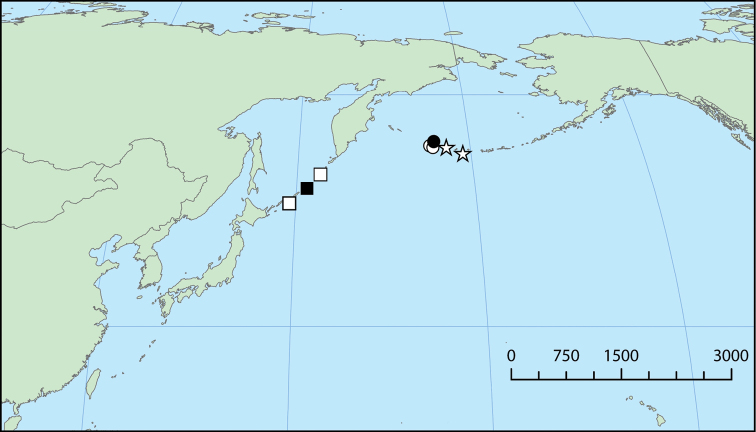
Distribution of *Pseudomohnia* gen. nov. in the North Pacific. Squares, *Pseudomohniakurilana* (Dall, 1913) comb. nov.; circles, *Pseudomohniarogerclarki* sp. nov.; stars, *Pseudomohnia* sp. Symbols with black fill are type localities. Scale in kilometers.

*Mohniakurilana* Dall, 1913 was described more than 100 years ago from deep waters off the Kuril Islands in the northwestern Pacific (Figs 1A, 2). In the absence of comparative material, the species has been retained in the genus *Mohnia* Friele, 1879, which is a member of the carnivorous neogastropod family Buccinidae (Kantor et al. 2021). Its shell and operculum bear a remarkable likeness to the type species, *Fususmohni* Friele, 1877, but are unlike those of other species currently assigned to the genus. Until recently, *Mohnia* was considered to be distributed in deep waters of the North Pacific and North Atlantic. However, recent revisions have seen many of its members allocated to closely related buccinoid genera, including *Fusipagoda* Habe & Ito, 1965, *Retifusus* Dall, 1916 and *Retimohnia* McLean, 1995 (McLean 1995; Kosyan and Kantor 2014, 2015, 2016; Kantor et al. 2021). This has left *Mohniakurilana* as the sole North Pacific member of the genus, otherwise represented by eight North Atlantic species (MolluscaBase 2021a).

Comparative material has remained exceedingly rare. Dried soft parts from the type lot of *Mohniakurilana*, as well as several specimens belonging to allied species collected in the Aleutian Islands in the 1990s, have enabled anatomical investigations for the first time, which allow its affinities to be reassessed.

## Materials and methods

Anatomical investigations of *Mohniakurilana* were based on dried soft parts in the type lot, USNM 205224. In addition, a fishery monitoring cruise conducted by the National Oceanic and Atmospheric Administration (**NOAA**) in the Aleutian Islands (Alaska) in 1997 yielded seven specimens of two allied species. This material was mostly dried, but included one fragmentary, alcohol-preserved body lacking a shell that at some point had been allowed to partially dry and that was broken at the base of the mantle cavity (LACM 1997-168.10). A second, highly fragmentary, alcohol-preserved body was used for a radula preparation (LACM 1997-156.7). Radulae were also prepared from several of the dried specimens by drilling a hole with a Dremel 8000-03 cordless rotary tool in the abapertural surface of the penultimate whorl and pushing the dried soft parts through the aperture with forceps.

Specimens were dissected under a Leica MZ 16.5 stereo microscope with camera lucida and stained with toluidine blue to enhance contrast. Radulae were tissue digested overnight in 100 µl of ATL lysis buffer (Qiagen, Inc.) containing ~ 50 µg of Proteinase-K, then sonicated and rinsed in de-ionized water (Holznagel 1997). Cleaned radulae were mounted on aluminum stubs using carbon adhesive tabs, coated with 25–30 nm gold/palladium (60/40), and imaged using an Apreo scanning electron microscope (FEI Company) at the National Museum of Natural History. Shells were photographed using a Canon EOS 50D camera and Canon MT-24EX macro twin light flash with a Canon EF 100 mm f/2.8 macro lens; protoconchs were photographed with a Canon MP-E 65 mm f/2.8 1–5X macro lens.

The classification used here follows Bouchet et al. (2017). See Discussion for the subfamily placement of the new genus *Pseudomohnia*.

Repositories:

**LACM**Natural History Museum of Los Angeles County, Los Angeles, California, USA;

**USNM**National Museum of Natural History, Smithsonian Institution (formerly U.S. National Museum), Washington DC, USA;

**ZIN**Zoological Institute of the Russian Academy of Sciences, St. Petersburg, Russia.

## Results

Anatomical investigations revealed the presence of a paucispiral operculum with an eccentric nucleus, foot with a deep propodial pedal gland and deep medial cleft bearing the opening of a metapodial pedal gland, short acrembolic proboscis, taenioglossate radula, well-developed mid-esophageal gland, glandular prostate, epiathroid nervous system with a long supra-esophageal connective, and numerous statoconia in the statocysts. The stomach and intestine contained abundant sponge spicules. This evidence indicates a placement in the Triphoroidea, a diverse superfamily specialized to feed on sponges, and to the Newtoniellidae in particular, a family distributed predominantly in cold, deep marine waters and frequently characterized by their dextral shell and taenioglossate radula (Bouchet and Warén 1993; Fernandes et al. 2015). *Mohniakurilana* is transferred to this family and is designated as the type species of a new genus, *Pseudomohnia* gen. nov. A second species from the Aleutian Islands is described as new, *Pseudomohniarogerclarki* sp. nov., distinguished by its multicuspid rachidian and narrowly turreted shell. Anatomical investigations indicate the presence of a third undescribed species, but we refrain from describing it pending the discovery of additional comparative material.

### Systematics

#### Class GASTROPODA Cuvier, 1795

**Superfamily TRIPHOROIDEA Gray, 1847**

##### Family NEWTONIELLIDAE Korobkov, 1955

###### Genus Pseudomohnia gen. nov.

Taxon classificationAnimaliaNeogastropodaBuccinidae

8B0FE460-8C9D-5F0A-A019-3644C77D42F5

http://zoobank.org/C28BBD54-8A95-4722-8F42-1A115E8533D4

Newtoniellidae gen. nov. pro “Mohnia” kurilana Dall, 1913: Sirenko, Kantor and Gulbin 2013: 156.

####### Type species.

*Mohniakurilana* Dall, 1913, here designated (Fig. 1A).

####### Description.

Shell dextral, thin, whitish in color, ~ 15–20 mm in adult shell length; whorls convex, suture deeply impressed, growth indeterminate. Protoconch large, multispiral, nucleus smooth, cancellate sculpture on subsequent whorls, transition to teleoconch gradual or indistinct. Teleoconch with spiral sculpture of fine cords and axial threads, often diminishing on body whorl and on base. Axis slightly gyrate, pervious; columellar plait lacking. Anterior canal short, slightly recurved. Operculum paucispiral with eccentric nucleus. Radula taenioglossate with small, concave rachidian, robust bicuspid lateral teeth, and slender marginal teeth with cylindrical shafts. Foot with deep propodial pedal gland and with metapodial pedal gland opening to deep medial cleft. Acrembolic proboscis short, salivary glands acinous, mid-esophageal gland well developed. Penis lacking. Nervous system epiathroid with long supra-esophageal connective.

####### Etymology.

In reference to the superficial similarity of the shell and its original placement in the genus *Mohnia* Friele, 1879 (Neogastropoda, Buccinoidea).

####### Distribution and ecology.

Known only from the Kuril and Aleutian Islands (Fig. 2) in 114–660 m, feeding on sponges.

####### Remarks.

The unique combination of shell and radula characters displayed by *Pseudomohnia* are unknown in the family and cannot be confused with any other genus. The recognition of *Mohniakurilana* as representing a new genus of Newtoniellidae had already been noted by Sirenko et al. (2013) based on as yet unpublished evidence provided in the present paper.

As documented in a number of newtoniellids, the presence of a large, ribbed protoconch with a gradual or indistinct transition between protoconch and teleoconch, despite being multispiral, points to a non-planktotrophic and intra-capsular mode of larval development (e.g., Marshall 1977; Bouchet and Warén 1993; Tsuchida and Sasaki 1998; Gofas 2003; Fernandes et al. 2015).

Little is known of the anatomy of newtoniellids, but that of *Pseudomohnia* compares favorably with Houbrick’s (1987) description of *Ataxocerithiumeximium* Houbrick, 1987 in the presence of a deep propodial pedal gland and a metapodial pedal gland opening to a deep medial cleft in the foot sole, a broad muscular snout, short acrembolic proboscis, well-developed mid-esophageal gland, and epiathroid nervous system with a long supra-esophageal connective.

###### Pseudomohnia kurilana

Taxon classificationAnimaliaNeogastropodaBuccinidae

(Dall, 1913) comb. nov.

A2AE0E17-F79C-5A6F-AA99-F4D0BBB2C333

**Figure 3.**
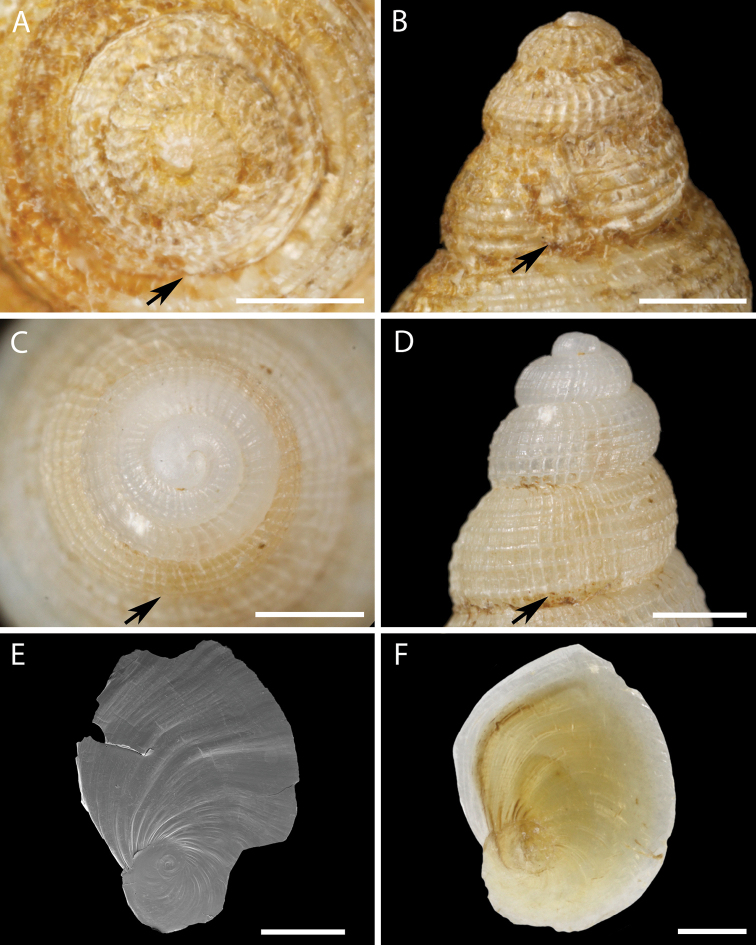
Protoconch and operculum morphology of *Pseudomohnia* gen. nov. **A, B, E***Pseudomohniakurilana* (Dall, 1913) comb. nov. Lectotype, USNM 205224 **C, D, F***Pseudomohniarogerclarki* sp. nov. Holotype, LACM 3776 **A, C** protoconch, apical view **B, D** protoconch, lateral view **E, F** Operculum. Black arrows indicate approximate position of inferred transition between protoconch and teleoconch. Scale bar: 1 mm.

**Figure 4.**
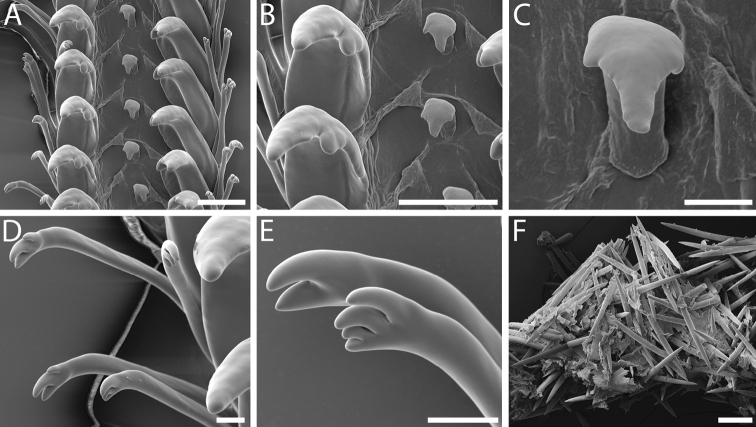
Radula morphology and gut contents of *Pseudomohniakurilana* (Dall, 1913) comb. nov., lectotype, USNM 205224 **A** radular ribbon **B** detail of rachidian and lateral teeth **C** detail of rachidian **D** detail of marginal teeth **E** detail of inner and outer marginal teeth **F** sponge spicules from gut. Scale bars: 20 µm (**C, D, E**); 100 µm (**A, B, F**).

**Figure 5.**
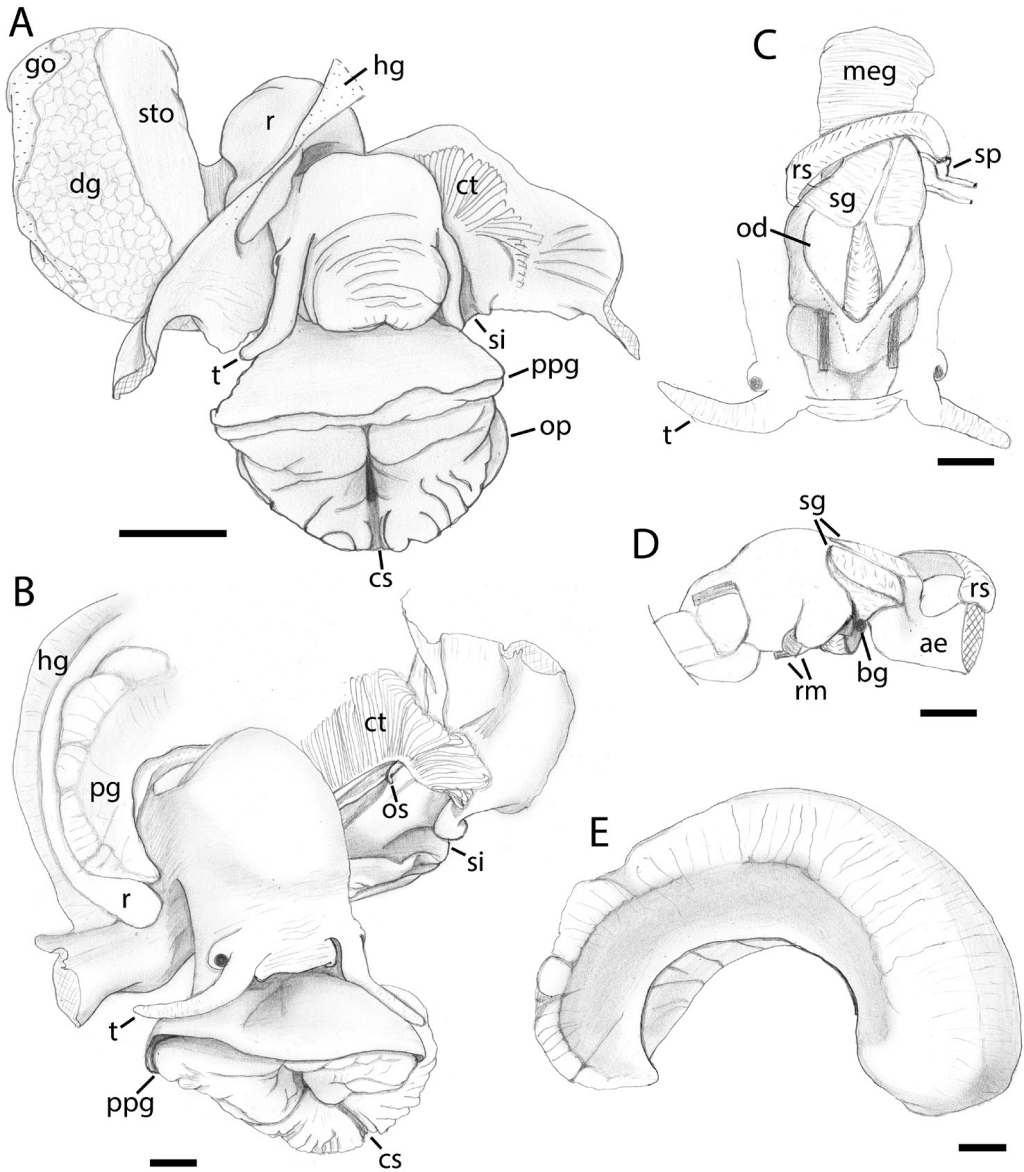
Anatomy of *Pseudomohnia* gen. nov. **A***Pseudomohniakurilana* (Dall, 1913) comb. nov., lectotype, USNM 205224 **B–E***Pseudomohnia* sp., LACM 1997-168.10 **A, B** external anatomy, dorsal view, mantle cavity opened via mid-dorsal incision **C** foregut, dorsal view **D** foregut, left-lateral view **E** pallial gonoduct, left-lateral view. Scale bars 1 mm. Abbreviations: ae, anterior esophagus; bg, buccal ganglion; cs, longitudinal cleft in foot sole; ct, ctendium; dg, digestive gland; go, gonad; hg, hypobranchial gland; meg, mid-esophageal gland; od, odontophore; op, opercular lobe; os, osphradium; pg, pallial gonoduct; ppg, propodeal pedal gland; r, rectum; rm, retractor muscles; rs, radular sac; sg, salivary gland; si, siphon; sp, supra-esophageal ganglion; sto, stomach; t, cephalic tentacle.

Figs 1A, B , 3A, B, E , 4 , 5A

Mohnia kurilana Dall, 1913: 503; Dall 1925: 21, pl. 34, fig. 1; Kosuge 1972, pl. 12, fig. 2 (Fig. 1A)
Mohnia kurilana : Golikov and Sirenko 1998: 112, fig. 7E; Kantor and Sysoev 2006: 187, pl. 93, figs J, J’ (Fig. 1B)

####### Type material.

***Lectotype*.** Kuril Islands • 13.40 mm in length; off Simushir Island; 46°42'N, 151°45'E; 229 fm [~ 419 m]; 24 June 1906; USFC steamer Albatross stn 4803; USNM 205224, here designated (Figs 1A, 3A, B, E, 4, 5A).

####### Other material.

Kuril Islands • 1 spm; near Iturup Island; 44°47.7'N, 148°55.5'E; 660 m; 27 July 1984; R/V Odyssey; B Sirenko leg.; ZIN 57494/1 (Fig. 1B); • 2 spms; Krusenstern Strait; 48°35.6'N, 153°54.8'E; 210 m; 9 October 1987; R/V Tikhookeanskiy; V Lukin leg.; ZIN 62774/2.

####### Description.

***Shell*.** Shell broadly turreted, spire angle ca. 42°, ~ 14 mm in adult shell length, consisting of approximately six, thin, convex whorls, separated by deeply impressed suture (Fig. 1A, B); growth indeterminate. Shell whitish, with thick, velvety periostracum. Larval shell non-planktotrophic, ~ 2.75 low, convex whorls, with smooth, blunt nucleus; well-defined opisthocyrt riblets and spiral threads producing cancellate sculpture on subsequent whorls. Inferred transition to teleoconch marked by change in orientation of axial sculpture and slight expansion in whorl diameter (Fig. 3A, B). Teleoconch with six to eight distinct, flattened, regular, spiral cords, separated by broader grooves, and which extend onto base. Spiral ornament crossed by variably developed, well separated, weakly prosocline axial threads and growth increments; axial threads becoming obsolete on body whorl. Aperture broad, outer lip thin, sharp. Axis slightly gyrate, pervious; columellar plait lacking. Anterior canal short, slightly recurved.

***Operculum*** [Lectotype]. Operculum thin, corneous, honey in color, thinning toward edges; paucispiral, nucleus eccentric, occupying ca. 31% of operculum length (Fig. 3E).

***Radula*** [Lectotype]. Radula taenioglossate (Fig. 4A). Rachidian small, concave, narrow, tapering slightly to chevron-shaped base (Fig. 4B, C). Cutting edge flaring slightly, bearing single, prominent, blunt cusp and smaller irregular denticle at outer edge on each side. Radular membrane diagonally creased between rachidian and lateral teeth of each row. Lateral teeth robust, broad, with smooth inner edge of shaft curving posteriorly; cutting edge with prominent, dagger-like, pointed cusp, occasionally with small, rounded accessory denticle at inner base, and with small, blunt inner cusp (Fig. 4A, B). Marginal teeth long, slender, with cylindrical shafts and slight constriction below claw-like tips; cutting edges of inner and outer marginal teeth bearing unequal numbers of short, curving, smoothly conical cusps, with three to four cusps on inner marginal teeth, and two to three on outer marginal teeth (Fig. 4D, E).

***Anatomy*** [Lectotype]. Foot elongate oval. Propodium large, triangular, presumably with deep, propodial groove (Fig. 5A), but depth could not be determined. Shallow furrow (= epipodial skirt) continuous with opercular lobe, evident along sides of foot sole, becoming obsolete in deep groove where propodium joins neck below snout. Foot sole divided longitudinally by deep medial cleft that deepens posteriorly before shallowing again along posterior quarter of sole. The presence and/or disposition of any glands could not be determined, although the epithelium in a broad swath on either side of the cleft is opaquely white and glandular in appearance.

Head with broad, muscular snout and long, tapering cephalic tentacles. Eyes conspicuous, on prominent ocular peduncles at outer bases of tentacles. Mantle edge smooth, with short siphon at left. Columellar muscle long, extending roughly one whorl to the level of mid-stomach. Mantle cavity one-half whorl in length. Mantle roof partially adhering to head and neck, not well preserved. Details of osphradium not readily observable. Ctenidium long, extending from siphon to base of mantle cavity. Hypobranchial gland well developed at left of rectum, releasing abundant mucus upon rehydration. Rectum broad, filled with sponge spicules, terminating in non-papillate anus well back from mantle edge. Pallial gonoduct presenting thin, non-glandular, open furrow below rectum. Penis lacking. Pericardial complex behind rear of mantle cavity compressed owing to retraction; details not observable. Bordered just behind by intestine.

Proboscis acrembolic, short. Jaws large, robust, dorsally flanking mouth. Buccal mass large with large odontophore occupying posterior two-thirds of buccal cavity behind jaws when retracted. Odontophore flattened, elongate, projecting upward from ventral posterior buccal mass, with flattened anterior end projecting against buccal roof behind jaws. Long, narrow, glandular subradular organ projecting between jaws from antero-ventral surface of odontophore. Moderately long radular ribbon emerging mid-ventrally near posterior end, embedded within acinous salivary glands posteriorly enclosing posterior buccal mass, and extending alongside anterior esophagus. Rather narrow mid-esophagus forming mid-esophageal gland with broad, glandular septae almost completely occluding lumen.

Stomach extending to ~ 1.5 whorls back from mantle edge, forming elongate chamber ~ 0.5 whorl in length, lying along left side of whorl ventrally surrounded by digestive gland. Stomach chamber broader posteriorly, narrowing anteriorly, filled with sponge spicules (Fig. 4F). Posterior esophagus entering stomach at left, near posterior quarter. Anterior end of stomach lying just behind reno-pericardial complex, large intestine curving right across body whorl, then turning anteriorly at base of mantle cavity on right side of body to form rectum. Digestive gland extending to anterior curve of intestine behind mantle cavity. Gonad dorsally overlying digestive gland at right of stomach.

####### Distribution and ecology.

Known only from the Kuril Islands (Fig. 2) in 210–660 m, feeding on sponges.

####### Remarks.

The original description stated that the type material was dredged by the U.S. Bureau of Fisheries steamer Albatross in 229 fathoms (~ 419 m) off the Kuril Islands. The original, handwritten accession ledger at the USNM indicates that it was collected off Simushir Island at station 4803, which was sampled on 24 June 1906, 46°42'N, 151°45'E.

Only limited morphological observations were possible given the size and condition of the dried soft parts but agree well with those obtained for *Pseudomohnia* sp. (see below). Although undeveloped, the pallial gonoduct appears to lack accessory sperm storage pouches (Fretter 1951; Houston 1985) and hence the dissected individual is inferred to be male. Details of the nervous system were not observable.

Analysis of the gut contents revealed the common occurrence of large (297 × 14 µm mean length × width) along with skinnier oxeas (248 × 6 µm) (Fig. 4F) which point toward a halichondriid sponge as the principal diet (Demospongiae, Suberitida, Halichondriidae) (K Ruetzler, pers. comm.).

###### Pseudomohnia rogerclarki sp. nov.

Taxon classificationAnimaliaNeogastropodaBuccinidae

5B78A912-0023-510A-870A-8929CB3A10A2

http://zoobank.org/B7AAEBD1-6DD5-4680-8B6F-71CD3754838E

**Figure 6.**
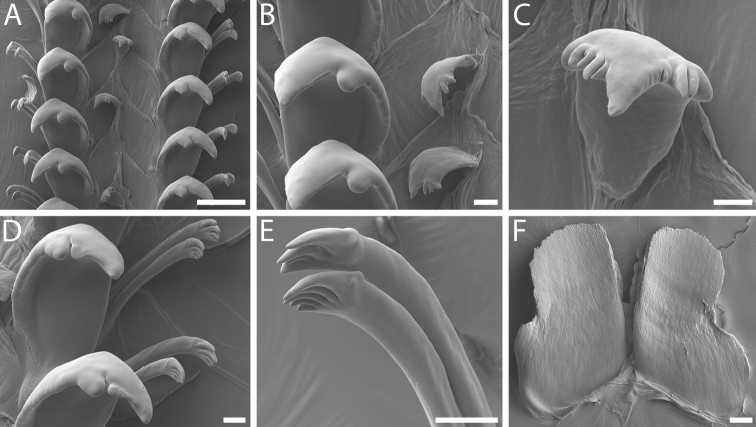
Radula and jaw morphology of *Pseudomohniarogerclarki* sp. nov., holotype, LACM 3776 **A** radular ribbon **B** detail of rachidian and lateral teeth **C** detail of rachidian **D** detail of lateral and marginal teeth **E** detail of inner and outer marginal teeth **F** jaw, inner surface. Scale bars: 10 µm (**C**); 20 µm (**B, D, E**); 100 µm (**A, F**).

Figs 1E–I , 3C, D, F , 6

####### Type material.

***Holotype*.** Aleutian Islands • 17.53 mm in length; Near Islands, north of Attu Island; 53°5.55'N, 173°43.46'E; 114 m; 4 August 1997; R/V Dominator stn 23-971-218; RN Clark leg.; LACM 3776 (ex LACM 1997-174) (Figs 1E, 3C, D, F, 6).

####### Other material.

Aleutian Islands • 2 spms; Near Islands, north of Attu Island; 53°5.55'N, 173°43.46'E; 114 m; 4 August 1997; R/V Dominator stn 23-971-218; RN Clark leg.; LACM 1997-174 (Fig. 1F, G); • 1 spm; Near Islands, south of Attu Island; 52°29.30'N, 172° 57.50'E; 166 m; 2 August 1997; R/V Dominator stn 23-971-210; RN Clark leg.; LACM 1997-163.21 (Fig. 1H); • 1 spm; Near Islands, south of Agattu Island; 52°13.50'N, 173°27.80'E; 166 m; 6 August 1997; R/V Dominator stn 23-971-229; RN Clark leg.; LACM 1997-165.20 (Fig. 1I).

####### Description.

***Shell*.** Shell narrowly turreted, spire angle ca. 30°, ~ 18 mm in adult shell length, consisting of approximately eight, thin, convex whorls, separated by deeply impressed suture (Fig. 1E–I); growth indeterminate. Shell whitish, with thick, velvety periostracum. Larval shell non-planktotrophic, ~ 3 elevated, constricted whorls, with smooth, blunt nucleus; well-defined opisthocyrt riblets and spiral threads producing cancellate sculpture on subsequent whorls. Prominent thread at shoulder and flattened subsutural ramp producing angulate appearance of first 1–1.5 whorls; gradually becoming more convex. Axial elements becoming more closely spaced toward teleoconch transition. Inferred transition to teleoconch marked by change in orientation of axial sculpture and slight expansion in whorl diameter (Fig. 3C, D). Teleoconch with seven to eight distinct, flattened spiral cords, somewhat irregular in width and spacing, and which extend onto base but may be less distinct. Spiral ornament crossed by variably developed, well separated, weakly prosocline axial threads and growth increments; axial threads obsolete on base. Aperture broad, outer lip thin, sharp. Axis weakly gyrate, pervious; columellar plait lacking. Anterior canal short, slightly recurved.

***Operculum*** [Holotype]. Operculum thin, corneous, honey in color, thinning toward edges; paucispiral, nucleus eccentric, occupying ca. 38% of operculum length (Fig. 3F).

***Radula*** [Holotype and LACM 1997-163.21]. Radular ribbon long, comprising 40 rows, ~ 3.7 mm in length, to 51 rows, ~ 4.4 mm in length (holotype). Radula taenioglossate (Fig. 6A). Rachidian small, concave, with sharp constriction below broad cutting edge, tapering to flat or pointed, narrow base. Cutting edge straight, bearing single, prominent, sharply pointed, triangular cusp and zero to four smaller, irregular, occasionally bifid denticles on each side (Fig. 6B, C). Radular membrane diagonally creased between rachidian and lateral teeth of each row. Lateral teeth robust, broad, with undulating inner edge of shaft curving posteriorly; cutting edge with prominent, dagger-like, pointed cusp, rarely with small, rounded accessory denticle at inner base, and with small, blunt inner cusp (Fig. 6A, B, D). Marginal teeth long, slender, with cylindrical shafts and constriction below claw-like tips; cutting edges of inner and outer marginal teeth bearing three to six elongate, curving, pointed cusps (Fig. 6E).

***Anatomy*** [Holotype and LACM 1997-163.21]. Jaws large, robust, oval-rectangular to L-shaped (holotype; Fig. 6F), partially connected across posterior midline via thin membrane, comprised of rectangular rods. Rectum filled with sponge spicules.

####### Etymology.

In honor of Roger N Clark, Associate in Malacology at the LACM, who collected the type material during a fishery monitoring cruise conducted by NOAA in the Aleutian Islands in 1997.

####### Distribution and ecology.

Known only from the Aleutian Islands (Fig. 2) in 114–166 m, feeding on sponges.

####### Remarks.

This species differs from *Pseudomohniakurilana* in having constricted and elevated early whorls with an early angulation that is somewhat variable among available specimens. The shell is more turreted, with a narrower spire angle, and the operculum has a slightly larger nucleus. The radula is distinguished by the multicuspid rachidian and a greater number of cusps on the marginal teeth.

A radula preparation showing the distinctive multicuspid rachidian that was photographed in July 2006 could not be located in the collections of the LACM (L Groves, pers. comm.). Notes in an unpublished draft for the Northeast Pacific Gastropod volume indicate that LACM 1997-165.20 was used for a radula preparation. This lot (Fig. 1I) contains a damaged shell with a broken operculum and dried soft parts lacking a head.

###### Pseudomohnia

Taxon classificationAnimaliaNeogastropodaBuccinidae

sp.

4E6C39F1-EBAE-5C71-B7F4-442F99FD7EFF

**Figure 7.**
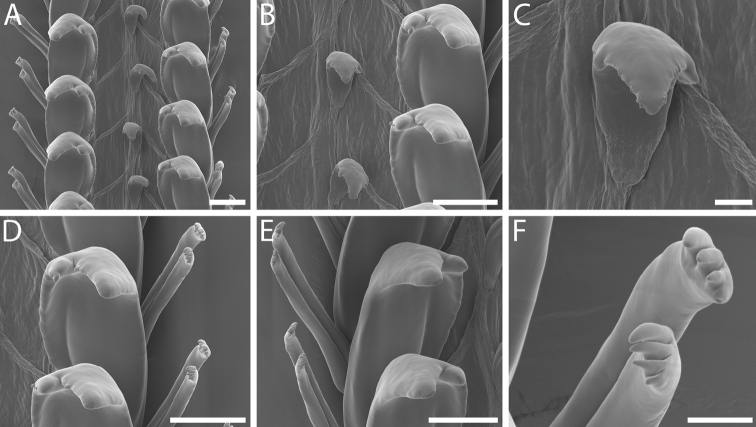
Radula morphology of *Pseudomohnia* sp., LACM 1997-168.10 **A** radular ribbon **B** detail of rachidian and lateral teeth **C** detail of rachidian **D, E** detail of lateral and marginal teeth **F** detail of inner and outer marginal teeth. Scale bars: 20 µm (**C, F**); 100 µm (**A, B, D, E**).

Figs 1C, D , 5B–E , 7

####### Material examined.

Aleutian Islands • 2 spms; Rat Islands, southwest of Amchitka Island; 51°27.70'N, 178°35.0'E; 384 m; 27 July 1997; R/V Dominator stn 23-971-181; RN Clark leg.; LACM 1997-156.7 (Fig. 1C, D); • 1 spm; southwest of Buldir Island; 52°18.50'N, 175°49.0'E; 325 m; 9 August 1997; R/V Dominator stn 23-971-243; RN Clark leg.; LACM 1997-168.10 (Figs 5B–E, 7).

####### Description.

***Shell*** [LACM 1997-156.7]. Shell broadly turreted, spire angle ca. 41°, ~ 24 mm in adult shell length, consisting of approximately eight, thin, convex whorls, separated by deeply impressed suture (Fig. 1C, D); growth indeterminate. Shell whitish, with thick, velvety periostracum. Larval shell non-planktotrophic, ~ 3 elevated, constricted whorls, with smooth, blunt nucleus; well-defined opisthocyrt riblets and spiral threads producing cancellate sculpture on subsequent whorls. Axial elements becoming more closely spaced toward teleoconch transition. Inferred transition to teleoconch marked by change in orientation of axial sculpture and slight expansion in whorl diameter. Teleoconch initially with six or seven thin spiral cords, somewhat irregular in width and spacing; cords becoming flatter, broader and less distinct on later whorls and on base and intercalated by additional cords. Spiral ornament crossed by variably developed, well separated, weakly prosocline axial threads and growth increments; axial threads becoming obsolete on body whorl. Aperture broad, outer lip thin, sharp. Axis weakly gyrate, pervious; columellar plait lacking. Anterior canal short, slightly recurved.

***Operculum*** [LACM 1997-156.7]. Operculum thin, corneous, honey in color, thinning toward edges; paucispiral, nucleus eccentric, occupying ca. 42% of operculum length.

***Radula*** [LACM 1997-168.10]. Radular ribbon comprising 37 rows, ~ 5.7 mm in length. Radula taenioglossate (Fig. 7A). Rachidian small, concave, with slight constriction below broad cutting edge, tapering to flat, narrow base. Cutting edge bearing single prominent, broadly triangular, bluntly pointed, finely serrated cusp, and single smaller, irregular denticle on each side (Fig. 7B, C). Radular membrane diagonally creased between rachidian and lateral teeth of each row. Lateral teeth robust, broad, with smooth inner edge of shaft curving posteriorly; cutting edge with prominent, dagger-like, pointed cusp and small, blunt inner cusp (Fig. 7B, D, E). Marginal teeth long, slender, with cylindrical shafts and constriction below claw-like tips; cutting edges of inner and outer marginal teeth bearing three to five elongate, curving, pointed cusps, somewhat angular in cross section (Fig. 7E).

***Anatomy*** [LACM 1997-168.10]. Foot elongate oval. Propodium large, crescent shaped, with deep, triangular propodial groove (Fig. 5B). Shallow furrow (= epipodial skirt) continuous with opercular lobe, evident along sides of foot, becoming obsolete in deep groove where propodium joins neck. Foot sole, particular that of metapodium, deeply wrinkled with many deep transverse grooves. Sole divided longitudinally by deep medial cleft. Rather large bilobed mesopodial pedal gland within foot below and in front of pedal ganglia on both sides of cleft, opening via large pore near center of sole.

Head with short, broad, muscular snout and long, tapering cephalic tentacles (Fig. 5B). Eyes conspicuous, on prominent ocular peduncles at outer bases of tentacles. Mantle edge smooth, with short, clearly defined siphon at left. Columellar muscle short, broad, extending roughly one-half whorl to base of mantle cavity. Ctenidium long, extending from siphon to base of mantle cavity, with long, narrowly triangular leaflets. Osphradium forming tall, narrow, undulating ridge, extending almost entire length of gill, from near anterior end almost to base. Hypobranchial gland well developed. Rectum broad, filled with sponge spicules, terminating in papillate anus near mantle edge at right. Rectum bordering pallial glandular gonoduct. Pallial gonoduct open for much of its length, lacking accessory pouches; thick, highly glandular tissue subdivided by deep transverse grooves (Fig. 5E). Penis lacking.

Proboscis acrembolic. Introvert rather short, muscular, oral tube not cuticularized. Jaws large, robust, surrounding anterior end of odontophore (Fig. 5C). Buccal mass large with long radular sac (Fig. 5C, D) emerging mid-ventrally near posterior end, continuing to right before arcing dorsally across anterior esophagus just behind buccal mass with posterior, weakly-bifid tip lying on left side of esophagus near supra-esophageal ganglion. Posterior buccal cavity with broad, deep, subtriangular, acinous salivary glands on either side of dorsal food groove. Anterior esophagus not cuticularized. Large mid-esophageal gland (Fig. 5C) with shallow, glandular septae and voluminous lumen, narrowing to posterior esophagus near end of mantle cavity, with ca. seven low, longitudinal folds.

Nervous system epiathroid. Circum-esophageal nerve ring surrounding anterior esophagus (Fig. 5C) just behind buccal apparatus. Nerve ring highly asymmetrical, with both cerebral ganglia lying on left side of esophagus; left cerebral ganglion below and slightly in front of right ganglion, joined by very short but distinct commissure. Buccal ganglia (Fig. 5D) joined by short commissure, lying on either side of posterior buccal mass at emergence of anterior esophagus, just below salivary glands. Small pleural ganglia lying immediately behind cerebral ganglia, separated by narrow constrictions. Long connective joining right pleural with supra-esophageal ganglion (Fig. 5C) at left side of cephalic hemocoel near tip of radular ribbon. Sub-esophageal ganglion lying below right side of anterior esophagus, separated from left pleural ganglion by slight constriction. Long, highly asymmetric connectives joining cerebral and pleural ganglia with pedal ganglia lying within foot at short distance anterior to cerebral ganglia. Small statocysts with numerous, tiny statoconia on postero-dorsal surface of pedal ganglia. Pedal ganglia joined by short, thick commissure.

####### Distribution and ecology.

Known only from the Aleutian Islands (Fig. 2) in 325–384 m, feeding on sponges.

####### Remarks.

Given the absence of accessory sperm storage pouches in the pallial gonoduct (Fretter 1951; Houston 1985), the dissected individual is inferred to be male.

The disposition of the remnants of the shell from LACM 1997-168.10 on which the anatomical observations were made is unknown and there is no known photograph (RN Clark, L Groves, pers. comm.). The anatomy and radula morphology show several differences compared to *Pseudomohniakurilana* and *P.rogerclarki* (see below). The two specimens in LACM 1997-156.7 (Fig. 1C, D) have a broader spire angle than *P.rogerclarki*, but share the constricted, elevated early whorls. The spiral sculpture of the teleoconch is less distinct and more irregular than in *P.kurilana* and *P.rogerclarki*. Fragmentary soft parts from one of the two specimens in LACM 1997-156.7 (Fig. 1D) produced a radula that we infer to be teratological, lacking a rachidian and bearing stunted marginal teeth with weakly lobed tips. The two available lots were collected in deeper waters (325–384 m) than *P.rogerclarki* (114–166 m). Given the fragmentary and incomplete information available, we cautiously conclude that the broad morph represented by the two specimens in LACM 1997-156.7 is conspecific with LACM 1997-168.10 and represents a third and undescribed species of *Pseudomohnia*. It is possible that the shells and the soft parts are not conspecific, and that the broad morph represents population variation or sexual dimorphism within the range of *P.rogerclarki*. Thus, we refrain from describing another species until additional comparative material becomes available.

To the extent that comparisons are possible, the anatomy of *Pseudomohnia* sp. agrees well with that of *P.kurilana*. The most conspicuous differences between the two concern details of the anterior alimentary system; specifically, the salivary glands appeared more irregular, the snout longer, the radular sac shorter, the mid-esophageal gland less developed, and the length of the introvert shorter in *P.kurilana*. The length of the introvert is known to vary within the family (Houbrick 1987; Golding et al. 2009), but that of *P.kurilana* also may have been incompletely retracted which would also explain the appearance of the snout. *Pseudomohnia* sp. is more similar to *P.kurilana* in morphology of the radula, but differs in the broader, more triangular and finely serrated central cusp of the rachidian and in the morphology of the marginal teeth which bear slightly fewer (two to four versus three to five), shorter, more smoothly conical cusps in *P.kurilana*. However, the range of values of marginal cusp counts overlaps in the two species and its significance could diminish with greater sampling. Like other triphoroideans, the nervous system is epiathroid with a long supra-esophageal connective, but differs in the presence of numerous, tiny statoconia in the statocysts rather than a single statolith (Risbec 1943).

## Discussion

In triphoroideans, the plesiomorphic taenioglossate condition has yielded an impressive diversity in tooth number and morphology possibly reflecting the diversity of their sponge hosts (e.g., Kosuge 1966; Marshall 1978, 1980, 1983, 1984; Bouchet 1985; Bouchet and Warén 1993; Nützel 1998; Fernandes and Pimenta 2019; Ponder et al. 2020). As far as is currently known, most newtoniellids have retained the taenioglossate configuration (Sars 1878; Thiele 1929; Powell 1951; Marshall 1978, 1980; Houbrick 1987; Bouchet and Warén 1993; Nützel 1998) apart from *Adelacerithium* Ludbrook, 1941 (Marshall 1984), *Sasamocochlis* Gründel, 1980 (Tsuchida and Sasaki 1998), and a species of *Trituba* Jousseaume, 1884 (Bouchet and Fechter 1981; as *Triforis*).

As noted by Ponder et al. (2008), triphoroideans have remained virtually untouched by comparative approaches. The anatomy of *Pseudomohnia* agrees well with what little has been described for members of the superfamily thus far (Risbec 1943; Fretter 1951; Kosuge 1966; Houston 1985), and for newtoniellids in particular (e.g., Houbrick 1987; Golding et al. 2009), and in their ecology as feeders on sponges. However, the family and subfamily classification currently in use (Bouchet et al. 2017) is based primarily on morphology of the shell (including protoconch) and radula; anatomical characters that might serve to distinguish family-level taxa have not been established (Ponder and Warén 1988) and the phylogenetic cohesiveness of the Ataxocerithiinae (which at present is a nomenclaturally unavailable name) and other currently recognized subfamilies remains unknown. This is an area ripe for testing within a molecular framework. Thus, we refrain here from making a more formal subfamily placement. That said, *Pseudomohnia* seems to bear the closest affinity to species currently classified in the Laeocochlidinae Golikov & Starobogatov, 1987 and particularly to *Laeocochlis* Dunker & Metzger, 1874. The latter is presently understood to contain a single Recent species, *Laeocochlissinistrata* (Nyst, 1835), distributed in offshore waters of the North Atlantic (Bouchet and Warén 1993; MolluscaBase 2021b). Both genera possess ribbed, multispiral protoconchs and development is inferred to be non-planktotrophic. The large teleoconchs are characterized by convex whorls separated by a deeply impressed suture and a dominant sculpture of spiral cords. The radulae are taenioglossate, with teeth that are solid and comparatively plain (Sars 1878; Thiele 1929; Powell 1951; Marshall 1978, 1980, 1984; Houbrick 1987; Bouchet and Warén 1993; Nützel 1998). The shell of *Pseudomohnia* differs in being dextral, smaller in length at adulthood (~ 15–20 mm vs. > 25 mm), more inflated with a broader spire angle, and with a less gyrate axis and a shorter, less distinct siphonal canal. The radula differs in the small, concave rachidian and slender marginal teeth with cylindrical shafts; the teeth of *Laeocochlis* are all unicuspid (Bouchet and Warén 1993: fig. 1281). The monotypic *Sasamocochlis*, also currently placed in the Laeocochlidinae (MolluscaBase 2021b), has a similarly large, sinistral shell with convex whorls and dominant spiral sculpture, and possesses a radula with unicuspid teeth, but lacks marginal teeth (Tsuchida and Sasaki 1998).

While showing some affinity to species currently classified in the Laeocochlidinae, the morphology of the radula and shell of *Pseudomohnia* presents many unique features previously undocumented in newtoniellids (Sars 1878; Powell 1951; Marshall 1978, 1980; Houbrick 1987; Nützel 1988; Bouchet and Warén 1993). Indeed, the unusual shell morphology has obscured its true affinities for over 100 years. Confirmation of its family placement will require molecular sequence data and the genus may merit recognition at a higher rank.

## Supplementary Material

XML Treatment for Pseudomohnia

XML Treatment for Pseudomohnia kurilana

XML Treatment for Pseudomohnia rogerclarki

XML Treatment for Pseudomohnia
